# Assessing treatment value under uncertainty: Measuring treatment value for a new treatment for cognitive impairment

**DOI:** 10.1002/alz.089925

**Published:** 2025-01-09

**Authors:** Jason Shafrin, Kyi‐Sin Than, Jaehong Kim, Jacob Fajnor, Elizabeth S Mearns, Stacey L Kowal, Thomas Majda, Jakub Hlavka

**Affiliations:** ^1^ FTI Consulting, Los Angeles, CA USA; ^2^ FTI Consulting, Washington DC, DC USA; ^3^ Genentech, Inc., South San Francisco, CA USA; ^4^ University of Southern California, Los Angeles, CA USA

## Abstract

**Background:**

The value payers and policymakers traditionally place on novel treatments for Alzheimer’s and other neurological diseases that cause cognitive impairment (CI) and dementia often ignores disease baseline severity. More patient‐centered economic evaluations—such as those estimating “insurance value”—incorporate the fact that individuals are typically risk‐averse and place high value on generous coverage of treatments in the event that they develop CI, such as severe dementia, later in life. This study aims to estimate this insurance value, accounting for risk aversion, relative to the value of the same treatment based on traditional payer cost effectiveness approaches.

**Method:**

A survey was administered to US residents aged ≥ 21 years. First, a multiple random staircase design was used to elicit respondents’ willingness to pay for insurance coverage of a hypothetical, novel treatment that delays progression from mild CI to dementia relative to best supportive care. The treatment’s “insurance value” was calculated as respondent’s stated willingness to pay less traditional value assessment estimates [i.e., the product of annual incidence (0.4%) and monetized, discounted value of quality‐adjusted life years (QALY) gained from treatment (0.652 QALYs x $100,000 per QALY)]. Risk aversion over quality‐of‐life outcomes was also estimated.

**Result:**

Among n = 295 respondents, 64.9% were female and average age was 51.1 (SD = 16.0). Willingness to pay for generous insurance coverage of a novel treatment delaying the onset of dementia was $646.88 per year compared to the value of the same treatment of $260.80 calculated under traditional cost‐effectiveness approaches. In other words, treatment value of novel treatments for CI were 2.5 times higher using the patient‐centered insurance value approach than using the traditional cost‐effectiveness methodology. Respondents were found to be risk averse (relative risk aversion = 1.49, 95% CI: 1.29, 1.68).

**Conclusion:**

Individuals place significant value on treatments that delay onset of cognitive impairments such as severe dementia. Traditional cost‐effectiveness may understate treatment value relative to more patient‐centered approaches that incorporate “insurance value”.

